# Forecasting individualized progression of Alzheimer’s disease using structural MRI and population spatiotemporal priors

**DOI:** 10.3389/fnagi.2026.1691084

**Published:** 2026-02-04

**Authors:** Yan Zhao, Tongtong Che, Xiuying Wang, Shuyu Li

**Affiliations:** 1 School of Health Science and Engineering, University of Shanghai for Science and Technology, Shanghai, China; 2 State Key Laboratory of Cognitive Neuroscience and Learning, Beijing Normal University, Beijing, China; 3 School of Computer Science, The University of Sydney, Sydney, NSW, Australia

**Keywords:** Alzheimer’s disease, generative artificial intelligence, progression prediction, spatiotemporal prior, voxel-wise change

## Abstract

**Objective:**

Alzheimer’s disease (AD), the most common neurodegenerative disorder, involves the progressive loss of vulnerable neurons. Tracking its progression via structural magnetic resonance imaging (sMRI), which captures subtle brain anatomical changes, is vital for advancing diagnosis and treatment. Although generative models show promise in simulating disease progression by forecasting future magnetic resonance imaging (MRI) sequences, generating high-quality MRI with faithful anatomical structures remains challenging.

**Methods:**

To narrow this gap, we proposed a progress map-guided generative adversarial network (pg-GAN) that leverages population-level longitudinal data to enhance individual-level prediction. First, progress maps were constructed by averaging intensity residuals between MRI scans acquired at different time points across a population, thereby preserving the comprehensive volumetric evolution of the brain over time. Then, the progress maps served as spatiotemporal priors and were embedded into a backbone generative adversarial network (GAN) via a proposed feature-wise fusion module (FFM) to predict future MRI for individuals.

**Results:**

We performed extensive experiments on 210 individuals with longitudinal MRIs from the Alzheimer’s Disease Neuroimaging Initiative (ADNI) dataset. The results demonstrated that our pg-GAN outperformed other conditioning models. The quantitative results showed that the normalized root mean squared error (NRMSE) decreased from 0.1623 to 0.1549, while the peak signal-to-noise ratio (PSNR) increased from 25.9353 dB to 26.3157 dB.

**Conclusion:**

Incorporating group-level progression priors into the generative model can significantly improve the accuracy and anatomical fidelity of predicted MRIs, enhance the visualization of disease progression at the voxel level, and advance the development of precision treatment for AD.

## 1 Introduction

Alzheimer’s disease (AD) is the most common cause of dementia, affecting an estimated 30 million people worldwide (Holtzman et al., 2011). AD gradually damages brain neurons, leading to progressive loss of memory and cognitive function. It often progresses through a transient clinical stage known as mild cognitive impairment (MCI) (Gaugler et al., 2022). AD-related brain morphological and anatomical changes, such as hippocampal atrophy and ventricular enlargement, can emerge 20 or more years prior to symptom onset (Younes et al., 2019). Thanks to advances in modern imaging technologies, these abnormal brain changes can be detected on structural magnetic resonance imaging (sMRI). Nowadays, magnetic resonance imaging (MRI) is extensively used in clinical practice and research due to its safety and accessibility (Burgos et al., 2021; Dickerson et al., 2011; Grueso and Viejo-Sobera, 2021; Myszczynska et al., 2020). Early intervention may delay the onset of AD. Hence, tracking brain changes is a crucial topic that can largely contribute to our understanding of AD progression.

Predicting disease progression is essential for early detection, precision diagnostics, individualized treatment, and even drug development for AD. Most neuroimaging-based studies have modeled disease progression as a classification (Abuhmed et al., 2021) or regression (Jiang et al., 2019; Li et al., 2019) problem. In classification modeling, a binary classifier is trained to distinguish between progressive MCI (pMCI) and stable MCI (sMCI). In regression modeling, a regressor is trained to estimate future cognitive scores or survival probabilities.

In fact, the progression patterns exhibit great heterogeneity due to the complex etiology and pathogenesis of AD. Even if two individuals yield the same classification or regression outcomes, their progression patterns may differ considerably. In addition, these modeling approaches are insufficient to explain why two individuals yield the same or different results or to interpret how the disease evolves. Therefore, a new paradigm is needed to visualize the dynamic spatiotemporal changes in brain images associated with disease progression. Tracking individual-specific, multi-session structural MRI over time can be a promising solution.

The rise of generative adversarial networks (GANs) (Goodfellow et al., 2014; Moulaei et al., 2024) has opened a promising avenue for disease progression prediction at the image level. In recent years, several GAN-based methods (Bernal et al., 2021; Fan et al., 2022; Ravi et al., 2022; Xia et al., 2021; Zhao et al., 2022; Zhao et al., 2021) have been proposed to forecast brain changes, directly predicting future brain structural MRI scans from baseline scans. This paradigm can support AD diagnosis and treatment with richer, more interpretable visual information. The first attempt to harness a GAN model for disease progression prediction at the image level was presented by Bowles et al. (2018), where the Wasserstein GAN was modified using an image arithmetic technique in the latent space to manipulate the hippocampus, temporal lobes, and lateral ventricles in structural MRI. Beyond linear modeling of particular brain regions, Ravi et al. (2022) developed a recursive GAN model to estimate future two-dimensional MRI slices in an end-to-end manner. Several GAN models have been developed to predict whole-brain MRI in three dimensions (Fan et al., 2022; Zhao et al., 2022). Recently, a few studies (Xia et al., 2021; Zhao et al., 2021) have incorporated auxiliary information into GAN models. For instance, Zhao et al. (2021) merged individual-specific attributes (e.g., age and sex) into the latent space of the generation model.

However, one-dimensional prior information is quite inadequate for the complex task. Therefore, our central goal is to capture more informative priors and enable them to effectively guide GAN models to produce more realistic future MRI scans. It is well known that brain changes vary considerably across different time intervals. For example, MRI over a 10-year interval exhibits greater atrophy than that over a one-year interval, not only in individuals at risk of AD but also in healthy aging populations. Nevertheless, no existing research has explored how to capture such spatiotemporal changes in MRI and harness this type of prior knowledge for image prediction.

**Figure 1:**
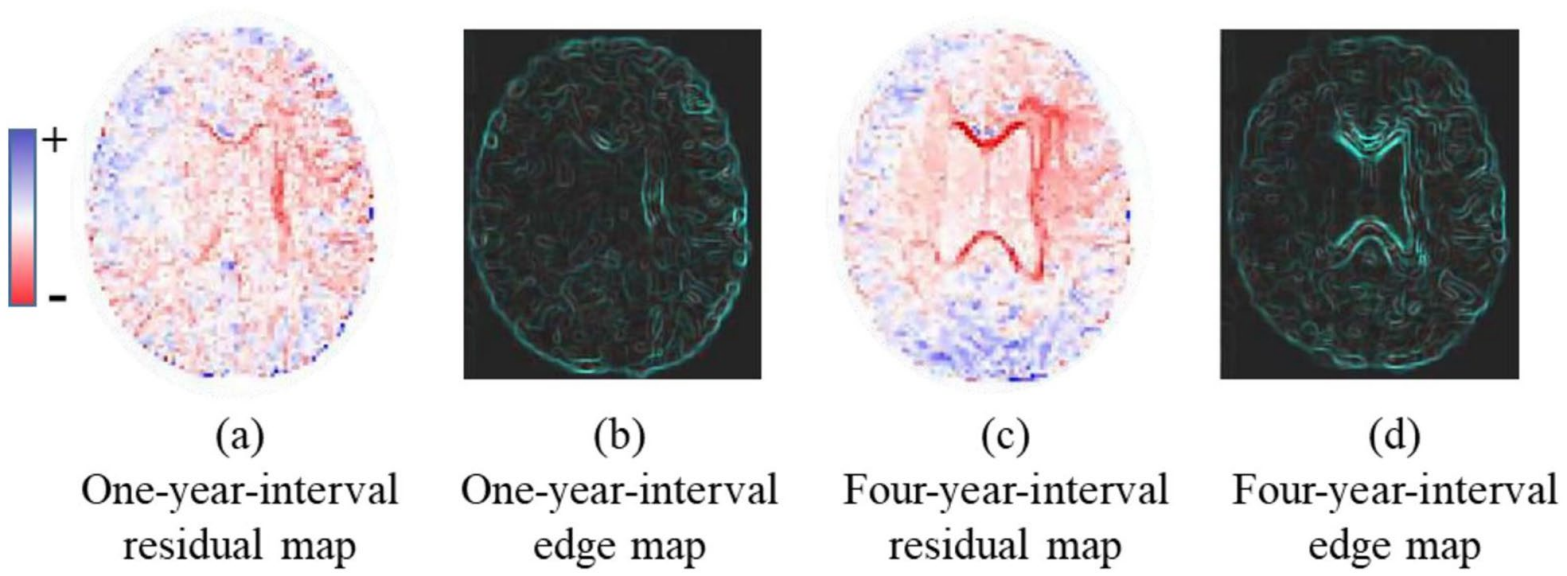
Progress map reveals brain spatiotemporal changes at the group level. **(a,c)** are the average residual map between structural MRIs at two timepoints, with 1-year and 4-year interval, respectively. **(b,d)** are the corresponding edge maps of **(a,c)**.

In this study, we introduce group-wise progress maps for the first time. As illustrated in Figure 1, progress maps can be established by averaging the intensity difference between the baseline and follow-up MRI scans, for example, the residual maps shown in Figures 1a,c. Alternatively, since image edge information (Yu et al., 2019) is critical for image synthesis, progress maps can be derived by applying the Sobel operator to the average residual maps, for example, the edge maps shown in Figures 1b,d. Then, we treat these progress maps as spatiotemporal priors and incorporate them into a backbone generative adversarial network.

Our main contributions are as follows:

- We hypothesize that group-wise brain MRI changes can prompt the prediction of subject-specific brain MRI scans.
- We characterize longitudinal brain changes using volumetric progress maps, constructed by averaging intensity differences between baseline and follow-up brain MRI scans across a population.
- We propose a progress map-guided generative adversarial network (pg-GAN), in which progress maps are used as spatiotemporal priors.
- We conduct extensive experiments on the Alzheimer’s Disease Neuroimaging Initiative (ADNI) dataset and demonstrate that spatiotemporal priors can improve the genuineness of predicted images.

## 2 Related research

Based on the dimensionality of input conditions, we categorized existing conditioning approaches into two types: the attribute-conditioned strategy and the map-conditioned strategy.

### 2.1 Attribute-conditioned strategy

The attribute-conditioned strategy is designed to embed one-dimensional information into artificial neural networks. The Feature-wise Linear Modulation (FiLM) layer (Perez et al., 2018) has been widely adopted in many vision-and-language tasks and extended by other conditional models. For example, FiLM was employed to incorporate modality factors, such as a non-spatial latent vector containing image modality information, into the decoder for cardiac image reconstruction (Chartsias et al., 2019). In the study by Dey et al. (2021), conditional face image templates were generated by learning feature-wise affine parameters with FiLM from input conditions, such as age and cohort. An MLP-based mapping network conditioned on a single input noise vector through FiLM was used to produce an implicit radiance field for 3D-aware image synthesis (Chan et al., 2021). FiLM performs an affine transformation based on task conditional vectors to adjust each image translation task (Takeda et al., 2021). To semantically edit an image with desired attributes (e.g., texture, color, and background) while preserving text-irrelevant content, a text-image affine combination module (Li et al., 2020) based on FiLM was developed. This module fuses text and image cross-modality representations by converting regional image features to scaling and bias values, which are then used to perform element-wise product and addition operations on the text features.

### 2.2 Map-conditioned strategy

Although FiLM has been used to enhance segmentation-relevant features by encoding anatomical information into a one-dimensional latent spatial factor (Cui et al., 2022), high-dimensional spatial data played a limited role in this framework, as only feature-wise affine transformations were considered. To fully exploit such critical spatial information, many map-conditioned models have been proposed that implement spatial-wise feature modulation. For instance, a spatial feature transform layer was developed to modulate intermediate features of a backbone super-resolution image generation model, with conditioning based on predefined semantic segmentation probability maps (Wang et al., 2018). A feature modulation module conditioned on the edge map prior was constructed to boost the reconstruction performance of the high-spatial-resolution hyperspectral image. The edge map was generated by applying the Sobel operator to the pre-trained feature maps of the high-spatial-resolution multispectral image (Zheng et al., 2021). Similarly, an edge guidance block (Tu et al., 2020) was designed to preserve spatial dimensions when incorporating edge prior knowledge by performing feature-wise manipulation and spatial-wise transformation on feature maps learned by a baseline salient object detection model. Moving beyond predefined segmentation maps, Li et al. (2021) integrated an edge detection network with a segmentation network and further designed a gated feature-wise transform layer to adaptively embed real-time, predicted hierarchical edge maps as guidance for semantic segmentation, which may help mitigate the impact of noisy edge information.

## 3 Methods

In the field of disease progression prediction via future MRI synthesis, attribute-conditioned methods (Chai et al., 2021; Ravi et al., 2022; Xia et al., 2021; Zhao et al., 2021; Jung et al., 2022; Dey et al., 2021) remain the primary approach employed to date. Compared to one-dimensional attributes, three-dimensional structural or anatomical information (Oh et al., 2022) can provide richer and more comprehensive guidance for models addressing such a complex task. However, no studies have yet harnessed such high-dimensional information to guide the generation of subject-specific future brain images. To address this critical research gap, the present study first characterized spatiotemporal changes in 3D brain structure over time and then leveraged this prior information to predict subject-specific future brain MRI scans. A core innovation of this study lies in the effective integration of 3D prior structural information into the prediction model—specifically, this integration enables the model to capture fine-grained anatomical changes associated with disease progression, which cannot be achieved by traditional attribute-conditioned methods.

Let $I_{\mathrm{bl}}\in R^{X\times Y\times Z}$ denote the brain MRI scanned at the baseline time point, with a spatial dimension of X×Y×Z. Let $I_{\mathrm{yx}}\in R^{X\times Y\times Z}$ denote the ground truth MRI scanned x years later, that is, the MRI scan at a follow-up visit. The spatiotemporal progress map is represented as $\;S_{\mathrm{yx}}\in R^{X\times Y\times Z}$, which captures the general structural changes of the brain between images at baseline and follow-up visits. Given $I_{\mathrm{bl}}$ and $S_{\mathrm{yx}}$, the future brain MR image ${\hat{I}}_{\mathrm{yx}}\in R^{X\times Y\times Z}$ is estimated to resemble the ground truth $I_{\mathrm{yx}}$. In short, the mapping function can be formulated as ${\hat{I}}_{\mathrm{yx}}=F(I_{\mathrm{bl}},S_{\mathrm{yx}})$.

### 3.1 Progress map construction

Inspired by Rachmadi et al. (2020), who quantified white matter hyperintensity (WMH) evolution by subtracting subjects’ baseline and follow-up irregularity maps to generate a two-dimensional probability or binary label map, we extended this concept to three dimensions to capture population-level brain structural changes.

Specifically, we first defined a group-level 3D progress map, which was constructed as a residual map by averaging voxel-wise intensity differences between baseline and follow-up 3D MRI scans across all individuals in the training set. Two such residual maps were produced: One for a one-year interval and another for a four-year interval. These maps encoded not only spatial but also temporal dynamic changes in brain MRI scans, as evidenced by the pronounced discrepancy between the one- and four-year patterns. Considering the average residual map may contain noisy or redundant information, we further derived an edge map by applying the Sobel operator to each residual map. The edge map may serve as an alternative form of the progression map.

Notably, all progress maps were built exclusively from training data; no individual in the test set was ever used, ensuring generalizability to unseen individuals.

### 3.2 Progress map-guided generator

**Figure 2:**
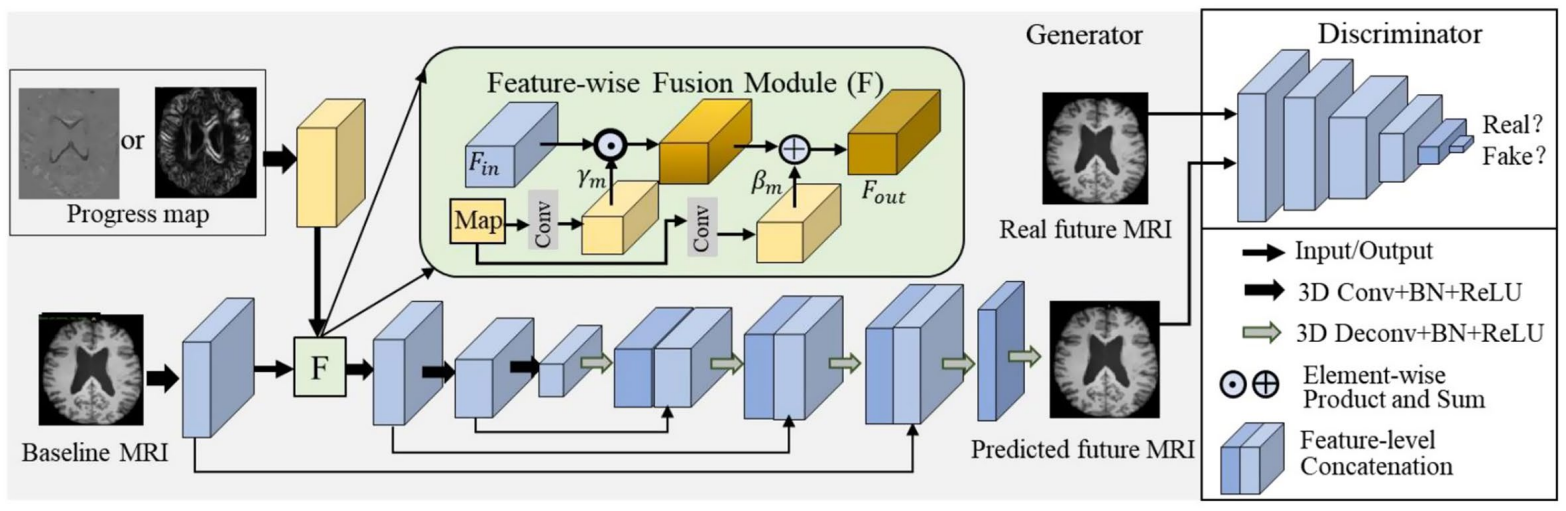
Overall network architecture for brain MRI prediction. It consists of a progression-map-guided generator and a discriminator. The progress map is inserted into the backbone network via the feature-wise fusion module (F), where the feature-wise fusion is conducted by modulating the baseline MRI features with a scaling parameter γ_m_ and a shifting parameter β_m._

The progress map was embedded into the network to guide future brain MRI prediction. The overall architecture is presented in Figure 2.

Motivated by the map-conditioned strategies reviewed in section 2.2, we inserted the volumetric progress map into the generator via a feature-wise fusion module (FFM). The feature-level fusion was performed by applying an affine transformation spatially to the first intermediate feature maps of the backbone generator, with the transformation parameters determined based on the progress map. In this way, the features of the baseline image and the progress map were merged.

The architecture of the feature-wise fusion module is illustrated in Figure 1. Specifically, the affine transformation was applied to the input feature maps $F_{\text{in}}$ using a scaling parameter $\gamma_{m}$ and a shifting parameter $\beta_{m}$. This parameter pair can adaptively influence the output feature maps $F_{\text{out}}$. We can formulate the above process as $F_{\text{out}}=\gamma_{m}(\mathrm{Map})$⊙$F_{\text{in}}⊕\beta_{m}$, where ⊙ and ⊕ represent the element-wise product and sum, respectively. The mapping from the progress map to the parameter pair can be represented by arbitrary functions. In this study, we used a convolutional layer (Conv), which was optimized together with the backbone network in an end-to-end manner. Consequently, spatiotemporal brain change information was embedded into the generator and guided its training through backpropagation.

The architecture of the feature-level progress map-guided generator was similar to that of the image-level one. The differences were mainly reflected in two aspects. First, the input and output feature maps of the first Conv were set to (1, 64) instead of (2, 64). Second, the progress map was fed into a Conv to learn the parameter pair used to transform the first intermediate feature maps in the generator via a feature-wise fusion module. The input and output feature maps, kernel size, and stride of this Conv were set to (1, 128), 4×4×4, and 2×2×2, respectively. The first 64 channels functioned as scaling parameters, and the latter 64 channels functioned as shifting parameters.

**Figure 3:**
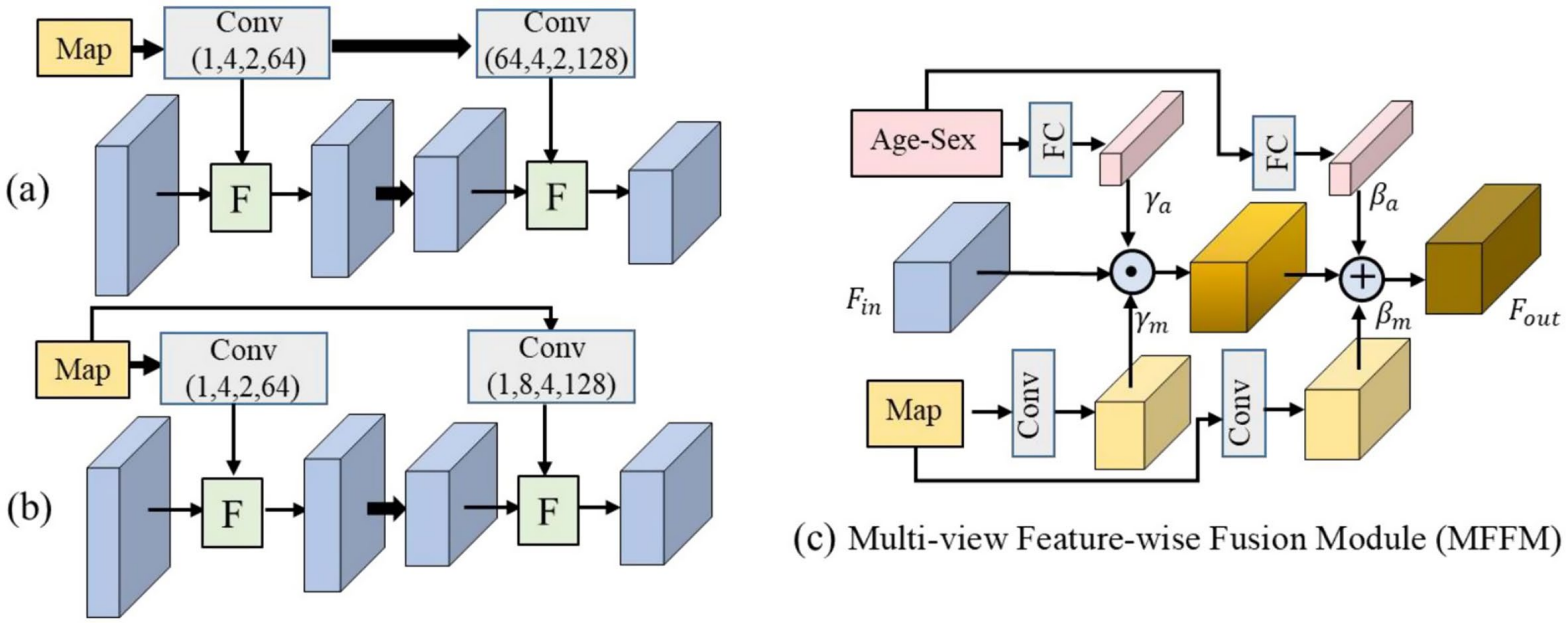
Other progress map embedding embodiments. **(a,b)** Illustrate two embedding embodiments that incorporate the features extracted from the progress map into the backbone generation model, where (1, 4, 2, 64) refers to the input feature channel, the kernel size, the stride, and the output feature channel of the convolution layer are 1, 4 × 4 × 4, 2 × 2 × 2, and 64, respectively. **(c)** Shows the multi-view feature-wise fusion module that incorporates both the features of attribute and progress map into the backbone generation model.

Furthermore, two or more feature-wise fusion modules can be incorporated into the model in two ways. Specifically, the input of the succeeding fusion module can be the feature maps extracted from the former layer, that is, in a parallel manner, as shown in Figure 3a. On the other hand, the input of the succeeding fusion module can be the feature maps directly extracted from the progress map, that is, in a cascade manner, as illustrated in Figure 3b.

The detailed architecture was based on the U-Net. There were four down-sampling blocks, followed by four corresponding up-sampling blocks. Each down-sampling block contained a Conv, a leaky ReLU layer, and a batch normalization layer. The kernel size and stride of each Conv were set to 4×4×4 and 2×2×2, respectively. The numbers of input and output feature maps from the first Conv to the last one were set to (2, 64), (64, 128), (128, 256), and (256, 512), respectively. Each up-sampling block contained a deconvolutional layer (Deconv), a batch normalization layer, and a leaky ReLU layer. In addition, the feature maps from each down-sampling block were copied and concatenated with the output feature maps of each corresponding up-sampling block. Therefore, the input and output feature maps from the first Conv and the subsequent ones were set to (512, 512), (512, 256), (256, 128), and (128, 64), respectively. Then, the output feature maps of the U-Net were fed into a Conv (the spatial filter is 1×1×1 with a stride of 1), ensuring that the image size of the predicted MRI scan was identical to that of the baseline MRI scan.

Moreover, a multi-view feature-wise fusion module (MFFM) was constructed, as depicted in Figure 3c, to incorporate both the progress map and attributes into the generator. Specifically, the subject attributes of age and sex were fed into two fully connected layers to learn a scaling parameter $\gamma_{a}$ and a shifting parameter $\beta_{a}$. In the meantime, the progress map was fed into the Conv to learn the scaling parameter $\gamma_{m}$ and the shifting parameter $\beta_{m}$. Then, the backbone feature maps $F_{\text{in}}$ were modulated by these parameters, which is formulated in Equation 1:


(1) $$F_{\text{out}}=(\gamma_{a}(\text{attr})+\gamma_{m}(\mathrm{Map}))⊙F_{\text{in}}⊕(\beta_{a}+\beta_{m})$$

where ⊙ and ⊕ represent the element-wise product and sum, respectively. In this way, the multi-view conditions, that is, subject attributes and the progress map, were embedded into the generation model.

### 3.3 Discriminator

Following GAN-based generative models (Ravi et al., 2022; Xia et al., 2021), we also incorporated adversarial learning into the prediction model. The discriminator learned to distinguish between real and synthetic MR images. The architecture of the discriminator consisted of four convolutional and leaky ReLU layers and two fully connected layers. The kernel size and stride of each convolutional layer were set to 4×4×4 and 2×2×2, respectively. The input and output feature maps from the first convolutional layer to the last layer were set to (1, 32), (32, 64), (64, 128), and (128, 256), respectively. The output nodes of the two fully connected layers were set to 1,000 and 1, respectively. Then, we adopted a sigmoid function to map the output of the final layer to the range of [0, 1], where the resulting value represents the probability of the input image being real or fake.

### 3.4 Loss function

Under the supervised learning paradigm, the loss of our proposed progress map-guided GAN model arose from image generation at the target time point and the discrimination process. Given a baseline image $I_{\mathrm{bl}}$ and a spatiotemporal progress map$\;S_{\mathrm{yx}}$, the progress map-guided generator synthesized a pseudo-image ${\hat{I}}_{\mathrm{yx}}=G(I_{\mathrm{bl}},S_{\mathrm{yx}})$ at a future time point. Meanwhile, ${\hat{I}}_{\mathrm{yx}}$ was fed into the discriminator to distinguish it from the ground truth image $I_{\mathrm{yx}}$. The joint training was optimized using the following objective function, shown in Equation 2.


(2) $$L_{\mathrm{adv}}(G,D)=E[\text{logD}(I_{\mathrm{yx}})]+E[\log(1-D(G(I_{\mathrm{bl}},S_{\mathrm{yx}})))]$$

In addition, we also leveraged the $L_{1}$ loss to capture the global appearance characteristics of the target image, which was formulated in Equation 3.


(3) $$L_{1}(G)=E[{\left\|I_{\mathrm{yx}}-G(I_{\mathrm{bl}},S_{\mathrm{yx}})\right\|}_{1}]$$

Therefore, the total objective function of the progress map-guided GAN model can be formulated Equation 4:


(4) $$L_{\text{total}}(G,D)=L_{\mathrm{adv}}(G,D)+\alpha L_{1}(G)$$

where α is a hyperparameter that balances $L_{\mathrm{adv}}$ and $L_{1}$.

## 4 Experiments

### 4.1 Dataset

The experiments were carried out on the Alzheimer’s Disease Neuroimaging Initiative (ADNI, adni.loni.usc.edu) dataset, a longitudinal, multi-site project aimed at the early detection of AD. Some individuals in the dataset underwent multiple observations over time. We used baseline (BL) to denote the first data collection. The sessions labeled “Y1” and “Y4” represent the data collected 1 and 4 years after the baseline, respectively. In this study, we utilized 210 participants with complete 3 T structural magnetic resonance imaging (sMRI) scans at BL, Y1, and Y4. The demographic details of these participants (108 male and 102 female) are provided in Table 1.

**Table 1:** Participants’ demographics at three time points.

Session	NC/MCI/AD	AGE	MMSE	ADAS-Cog
Baseline	76/134/0	71.32 ± 7.19	28.47 ± 1.60	7.81 ± 3.87
Year 1	81/119/10	72.34 ± 7.16	28.10 ± 2.03	7.29 ± 4.51
Year 4	86/87/37	75.43 ± 7.15	27.06 ± 3.83	9.38 ± 7.68

NC, MCI, and AD denote normal control, mild cognitive impairment, and patient with Alzheimer’s disease; MMSE and ADAS-Cog denote the mini–mental state examination score and Alzheimer’s Disease Assessment Scale-Cognitive Subscale, shown in a mean ± standard deviation format.

### 4.2 Experimental details

All sMRI volumes were first skull-stripped using the VBM toolbox^1^ and linearly aligned to MNI 152 space using FSL.^2^ Each image was centrally cropped to a size of 180×196×180 to remove the background and down-sampled by a factor of 2 to reduce GPU memory requirements. Then, we normalized the intensities of the brain image to the [0, 1] range using min-max normalization and subsequently rescaled to [−1, 1]. The processed images were randomly divided into training, validation, and test sets (170:20:20). We used the training set to construct the progress map to maintain the independence of the validation and test data. A total of four progress maps were built, including a one-year-interval residual map, a four-year-interval residual map, and their corresponding edge maps.

All experiments were conducted using TensorFlow^3^ with an NVIDIA Tesla P40 24 GB GPU. The progress map-guided GAN was trained using the Adam optimizer, with an exponential decay rate of 0.5 for the first moment and a mini-batch size of 4. The learning rate of both the generator and discriminator was set to 0.0001. The hyperparameter α was empirically set to 60. Training was stopped if the validation loss did not improve for 10 consecutive epochs.

### 4.3 Evaluation metrics

We used the peak signal-to-noise ratio (PSNR), normalized root mean squared error (NRMSE), and structural similarity (SSIM) to quantitatively evaluate the quality of the predicted future brain MR images (Sara et al., 2019; Zhan et al., 2022), calculated with scikit-image API.^4^ To be specific, these metrics can be computed in Equations (5–7):


(5) $$\text{PSNR}=10\times \log_{10}\frac{A*\mathrm{MA}X^{2}}{{\left\|I_{\mathrm{yx}}-{\hat{I}}_{\mathrm{yx}}\right\|}^{22}}$$

(6) $$\text{NRMSE}=\sqrt{\frac{{\left\|I_{\mathrm{yx}}-{\hat{I}}_{\mathrm{yx}}\right\|}^{22}}{{\left\|{\hat{I}}_{\mathrm{yx}}\right\|}^{22}}}$$

(7) $$\text{SSIM}=\frac{\left(2\mu_{I_{\mathrm{yx}}}\mu_{{\hat{I}}_{\mathrm{yx}}}+C_{1})(2\delta_{I_{\mathrm{yx}}{\hat{I}}_{\mathrm{yx}}}+C_{2}\right)}{\left(\mu_{I_{\mathrm{yx}}}^{2}+\mu_{{\hat{I}}_{\mathrm{yx}}}^{2}+C_{1})(\delta_{I_{\mathrm{yx}}}^{2}+\delta_{{\hat{I}}_{\mathrm{yx}}}^{2}+C_{2}\right)}$$

where MAX is the maximum intensity range of the ground truth $I_{\mathrm{yx}}$ and the predicted image ${\hat{I}}_{\mathrm{yx}}$; A represents the total number of voxels in the image. μ and δ are the mean and variance values of the image. $\delta_{I_{\mathrm{yx}}{\hat{I}}_{\mathrm{yx}}}$ is the covariance between images. $C_{1}$ and $C_{2}$ are two positive constants to avoid a null denominator. Theoretically, higher PSNR and SSIM values, along with lower NRMSE values, indicate better prediction performance.

## 5 Results

We evaluated our progress map-guided GANs on three tasks: (1) near-term prediction—synthesizing the 3D MRI 1 year after baseline; (2) long-term prediction—synthesizing the MRI 4 years after baseline; and (3) multi-term prediction—producing both one- and four-year follow-up images in a single forward pass. To quantify the benefits of incorporating the progress map, we compared the proposed model with three families of conditional GANs.

Baseline MRI-only GANs: We used the general conditional GAN model for image-to-image translation (Isola et al., 2017) to predict future brain images, feeding only the baseline image, without any auxiliary input. In addition, to investigate the effectiveness of the self-attention mechanism, we embedded the classical convolutional block attention module (CBAM) (Woo et al., 2018) into the conditional GAN model to adaptively learn features along both the channel and spatial dimensions, hereafter referred to as CBAM-GAN.

Attribute-conditioned GANs: We embedded one-dimensional attributes using two schemes. Following Xia et al. (2021) and Zhao et al. (2021) who concatenated the attribute vector with the bottleneck vector, we introduced the sex and the target age of the subject to the GAN model via feature concatenation; the model is referred to as mi-GAN. Following Dey et al. (2021), we also incorporated the attributes into the GAN model via feature-wise fusion, in which three FiLM layers were employed to perform feature transformation on the first three feature maps of the backbone network; this model is hereafter referred to as FiLM-GAN.

Pg-GANs: Either the residual map or the edge map served as the progress map. The pg-GANs merged the brain’s spatiotemporal change information with the baseline image using two strategies: Direct image-level concatenation or feature-level fusion. As a result, four models were constructed.

### 5.1 Performance of near-term MRI prediction

We first evaluated pg-GANs on the near-term prediction task. The quantitative comparison results are reported in Table 2, where the best value is in bold and an asterisk (*) denotes a significant difference (paired *t*-test, *p* < 0.05). As shown, the results achieved by our pg-GANs were better than the results of general conditional GANs and attribute-conditioned GANs across all evaluation metrics. For example, compared to the baseline GAN, the average residual map-guided GAN with image-level concatenation improved the NRMSE, SSIM, and PSNR by 0.0018, 0.0116, and 0.1403 dB, respectively. Although the CBAM-GAN and attribute-conditioned GANs (mi-GAN and FiLM-GAN) achieved some improvements, their results were still inferior to our progress map-guided GANs. For instance, the PSNR obtained by the edge map-guided GAN via feature-level fusion significantly increased by 0.1809 dB, 0.3650 dB, and 0.1184 dB. Based on the same progress map (residual map or edge map), feature-level fusion was better than direct image-level concatenation. However, based on the same guiding strategy (image-level concatenation or feature-level fusion), the paired *t*-test results showed that there was no significant difference between GANs conditioned on these two different progress maps. This may be because the features extracted from these two maps were similar, causing the model to primarily focus on the information that showed distinct brain changes.

**Table 2:** Performance of near-term brain MRI prediction.

BL → Y1		NRMSE ↓	SSIM ↑	PSNR ↑
[Image]	GAN	0.1524 ± 0.0539*	0.9244 ± 0.0422*	26.5888 ± 2.6310*
	CBAM-GAN	0.1525 ± 0.0539*	0.9349 ± 0.0422*	26.6081 ± 2.7099*
[Image, attribute]	Feature concatenation (mi-GAN)	0.1550 ± 0.0528*	0.9354 ± 0.0413	26.4240 ± 2.5665*
	Feature fusion (FiLM-GAN)	0.1517 ± 0.0552*	0.9359 ± 0.0424*	26.6706 ± 2.7357*
[Image, residual map]	Image-level concatenation	0.1506 ± 0.0553*	0.9360 ± 0.0418*	26.7291 ± 2.7162*
	Feature-level fusion	0.1501 ± 0.0539	0.9366 ± 0.0412	26.7416 ± 2.6572
[Image, edge map]	Image-level concatenation	0.1506 ± 0.0553*	0.9360 ± 0.0417	26.7278 ± 2.7122*
	Feature-level fusion	**0.1495** ± 0.0544	**0.9366** ± 0.0416	**26.7890** ± 2.7101

The results are shown in a mean ± standard deviation format. “BL→Y1” denotes the prediction of the 1-year follow-up image, with the input shown in the square bracket, including the baseline image and other conditions. ↓ means the lower, the better. ↑ means the higher, the better. Boldface highlights the best performance. An asterisk (*) denotes statistical significance (*p* < 0.05).

**Figure 4:**
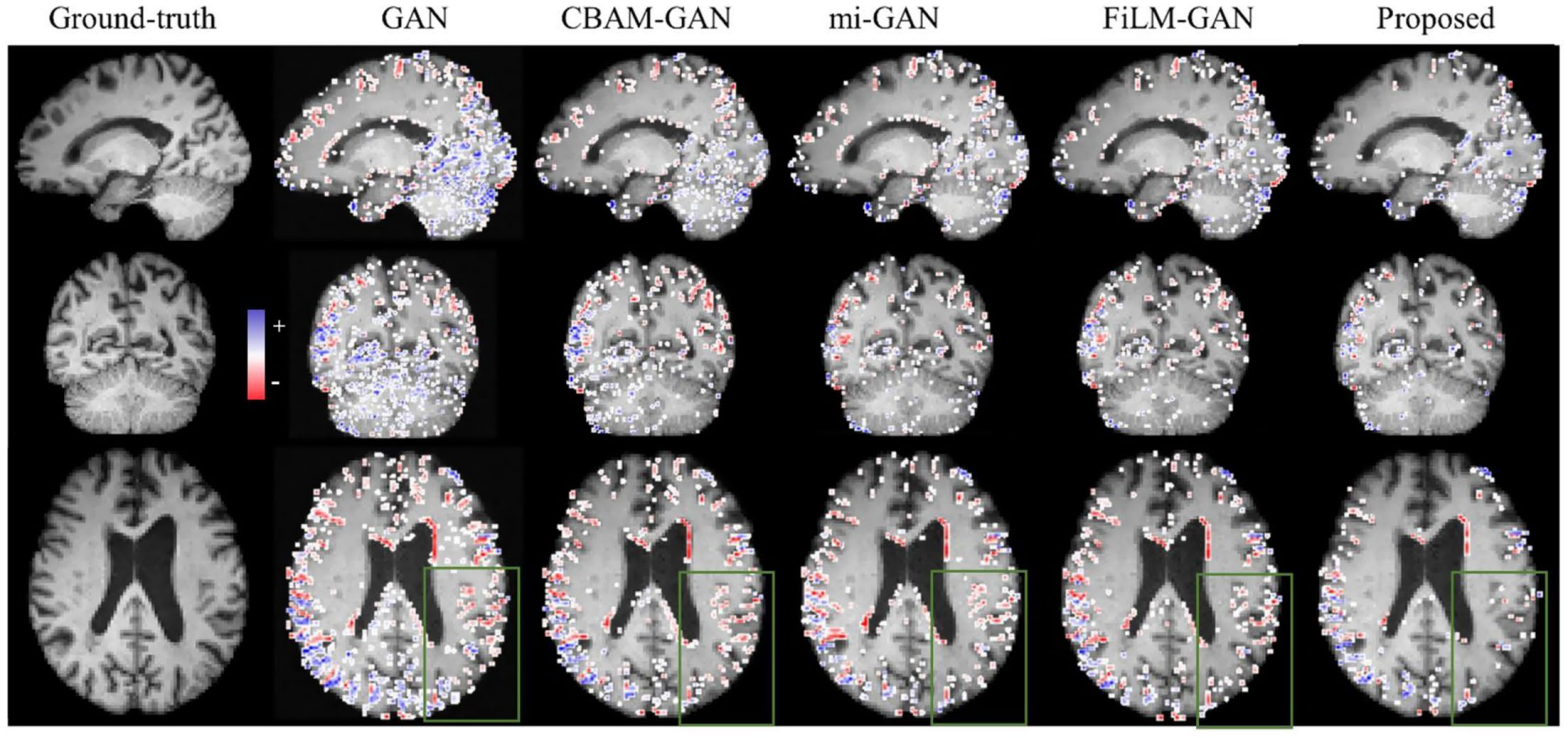
The comparison of error maps between ground-truth image and predicted future image within one-year interval along sagittal, coronal, and axial views. The blue dot means the predicted intensity of one voxel is higher than the real value. The red dot means the predicted intensity of one voxel is lower than the real value. More dense the dots, less faithful the image.

Although pg-GANs achieved better performance in visual effects, such as edge details, the differences between the images synthesized by different GANs were still subtle and not easily discernible. Therefore, we further provided error maps (Predicted Image–Ground truth) for a more intuitive check. Figure 4 illustrates the qualitative comparison results for a representative participant (PTID: 035_S_4082). The three rows show the 31st, 29th, and 43rd slices from the sagittal, coronal, and axial views, respectively. In general, the proposed edge map-guided GAN using feature-level fusion yielded higher-quality results (i.e., with a sparser error distribution) than other GANs, especially in the regions shown in the green box.

### 5.2 Performance of long-term MRI prediction

We then performed experiments to predict future brain MR images 4 years after baseline. Table 3 lists the quantitative comparison results. As shown, our proposed GAN—the edge map-guided GAN using feature-level fusion—still ranked first with an NRMSE of 0.1602, a SSIM of 0.9315, and a PSNR of 25.9160 dB, demonstrating the effectiveness of our progress map-guided GAN. For example, the PSNR exceeded that of the GAN, CBAM-GAN, mi-GAN, and FiLM-GAN models by 0.0248 dB, 0.2214 dB, 0.1868 dB, and 0.2454 dB, respectively.

**Table 3:** Performance of long-term brain MRI prediction.

BL → Y4		NRMSE ↓	SSIM ↑	PSNR↑
[Image]	GAN	0.1604 ± 0.0286	0.9275 ± 0.0262*	25.8912 ± 1.5008
	CBAM-GAN	0.1642 ± 0.0294*	0.9303 ± 0.0276*	25.6946 ± 1.5454*
[Image, attribute]	Feature concatenation (mi-GAN)	0.1636 ± 0.0297*	0.9306 ± 0.0269	25.7292 ± 1.5176*
	Feature fusion (FiLM-GAN)	0.1647 ± 0.0306*	0.9308 ± 0.0269*	25.6706 ± 1.5032*
[Image, residual map]	Image-level concatenation	0.1609 ± 0.0297	0.9314 ± 0.0279	25.8706 ± 1.5526
	Feature-level fusion	0.1603 ± 0.0304	0.9315 ± 0.0282	25.9167 ± 1.6087
[Image, edge map]	Image-level concatenation	0.1610 ± 0.0297	0.9314 ± 0.0278	25.8702 ± 1.5523
	Feature-level fusion	**0.1602** ± 0.0302	**0.9315** ± 0.0282	**25.9160** ± 1.5982

The results are shown in a mean ± standard deviation format. “BL→Y4” denotes the prediction of the four-year follow-up image, with the input shown in the square bracket [], including the baseline image and other conditions. ↓ means the lower, the better. ↑ means the higher, the better. Boldface highlights the best performance. An asterisk (*) denotes statistical significance (*p* < 0.05).

**Figure 5:**
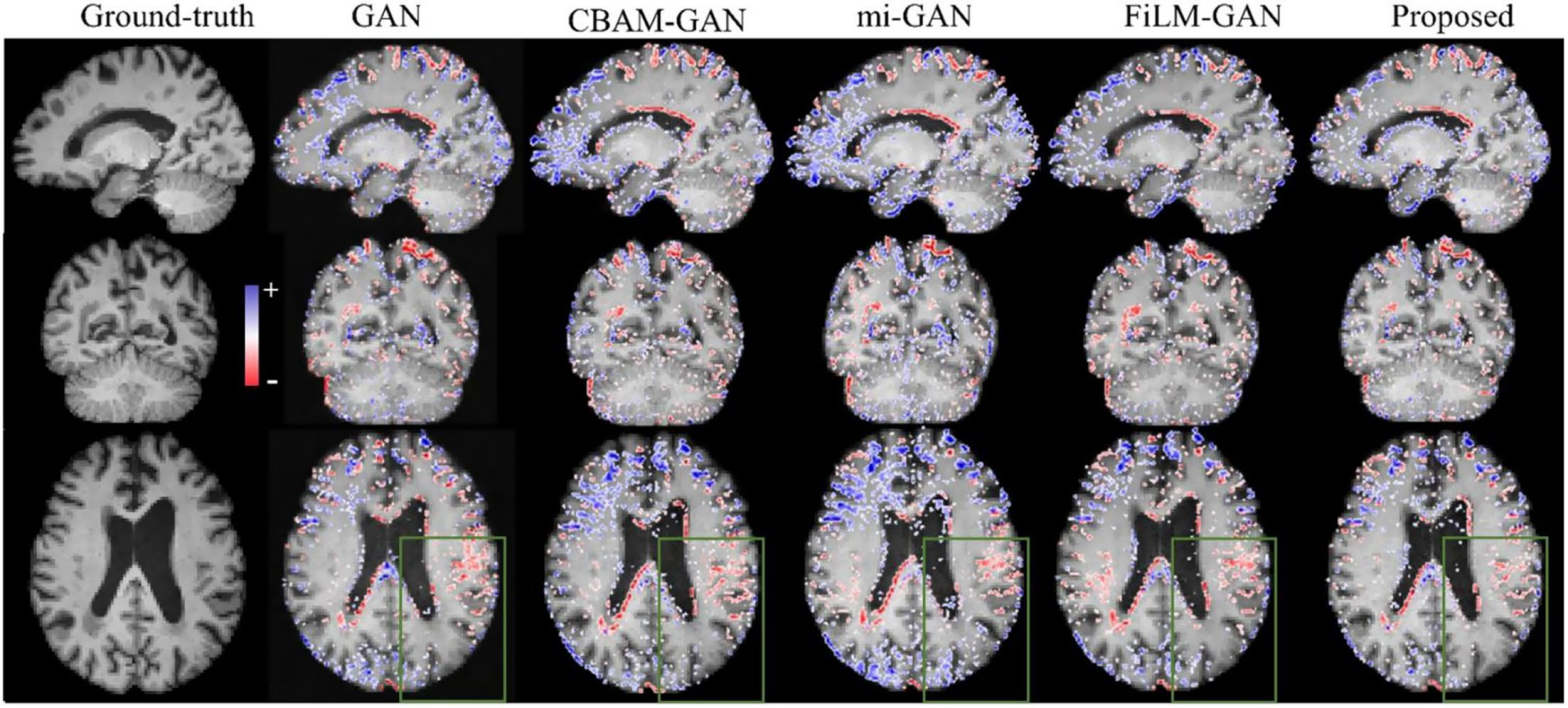
The comparison of error maps between ground-truth and predicted future images within four-year interval along sagittal, coronal, and axial planes. The blue dot means the predicted intensity of one voxel is higher than the real value, while the red dot means lower. Sparser the dots, more genuine the predicted image.

Furthermore, we visualized the predicted images for a representative participant 4 years after baseline, along with the corresponding error maps, in Figure 5. The three rows display the 31st, 29th, and 43rd slices in the sagittal, coronal, and axial views, respectively. Overall, the error map of the edge map-guided GAN with feature-level fusion was the least dense, confirming its clear advantage over competing methods. Within the green-boxed region, for example, our model reproduces cortical boundaries more accurately. Nevertheless, some areas—especially the cortical ribbon—remained slightly blurred and warrant further refinement. Errors were also noticeably larger than those in the one-year prediction, likely because long-term structural changes were both greater and more complex.

### 5.3 Performance of multi-term MRI prediction

In this subsection, we conducted multi-term prediction, that is, generating subject-specific brain MR images at two time points with a single training session, to further investigate the generalization ability of our method. Given the target attribute or progress map, GANs synthesized the corresponding MRI sequences. Specifically, given the input of the target age one year later, the attribute-conditioned GANs (mi-GAN and FiLM-GAN) yield the predicted image of that future stage; when a four-year progress map is provided as input, pg-GAN produces the corresponding long-term prediction image. The quantitative comparison results for the different GAN models are provided in Table 4. As shown, the NRMSE decreased from 0.1623 (mi-GAN) and 0.1580 (FiLM-GAN) to 0.1549, while the PSNR increased from 25.9353 dB (mi-GAN) and 26.1184 dB (FiLM-GAN) to 26.3157 dB. Paired *t*-tests confirmed that the proposed pg-GANs significantly outperformed the attribute-conditioned GANs (*p* < 0.05), demonstrating that the progress map materially improves long-term image prediction.

**Table 4:** Performance of multi-term brain MRI prediction.

BL → Y1&Y4		NRMSE ↓	SSIM ↑	PSNR ↑
[Image, attribute]	Feature concatenation (mi-GAN)	0.1623 ± 0.0447*	0.9321 ± 0.0365*	25.9353 ± 2.2742*
	Feature fusion (FiLM-GAN)	0.1580 ± 0.0402*	0.9336 ± 0.0344*	26.1184 ± 2.0613*
[Image, residual map]	Image-level concatenation	0.1554 ± 0.0421	0.9341 ± 0.0350*	26.3004 ± 2.1885
	Feature-level fusion	**0.1547** ± 0.0421	0.9347 ± 0.0346	**26.3339** ± 2.1630*
[Image, edge map]	Image-level concatenation	0.1554 ± 0.0422	0.9341 ± 0.0350	26.3018 ± 2.1895
	Feature-level fusion	0.1549 ± 0.0418	**0.9348** ± 0.0344	26.3157 ± 2.1450

The results are shown in a mean ± standard deviation format. “BL→Y1&Y4” denotes the prediction of both one-year and four-year follow-up images, with the input of the baseline image and other conditions. ↓ means the lower, the better. ↑ means the higher, the better. Boldface and an asterisk (*) denote the best performance and statistical significance, respectively (*p* < 0.05).

## 6 Discussion

Identifying patients who are susceptible to AD or experiencing AD progression is crucial for guiding treatment and developing preventive/therapeutic strategies. Nevertheless, despite extensive clinical and scientific efforts, predicting AD progression remains a significant challenge. Focusing on image-level disease progression prediction, this study makes three key contributions: (1) incorporating population-level spatiotemporal brain changes into AD progression modeling; (2) characterizing longitudinal brain changes using progress maps; and (3) proposing a pg-GAN that incorporates progress maps as spatiotemporal priors.

To capture longitudinal anatomical changes in brain MRI, an average residual map was constructed by averaging the residual intensity maps between subject-specific baseline and follow-up images. Since residual maps may contain excessive contextual noise, the Sobel operator was applied to generate an average edge map, which emphasizes the primary changes and provides sparse yet focused information. Both maps serve as effective progress maps. The proposed pg-GAN outperformed GANs using only baseline images and attribute-conditioned GANs, confirming that progress maps supply valuable auxiliary information for image prediction. Notably, the edge map-guided GAN achieved superior performance compared to the residual map-guided counterpart, and feature-wise fusion outperformed image-level concatenation in most cases.

Beyond monitoring AD progression using generated structural MRI (sMRI) scans, the proposed method facilitates various neuroimaging-based longitudinal analyses in brain development, aging, and scenarios requiring follow-up images for clinical decision-making. Potential applications include predicting the progression of osteoporosis, cardiovascular diseases, diabetes, and their related complications.

This study offers valuable insights into disease progression prediction. However, it is essential to acknowledge the study’s limitations. (1) The brain anatomical change pattern may be heterogeneous due to the complicated progression of AD. Therefore, the progress maps established in this study may be too coarse. More refined progress maps—such as group-specific maps for subpopulations, for example, male individuals aged 60–65 years—could be elaborately constructed if the sample size is sufficiently large. (2) We predicted future MRI scans at one- and four-year intervals. Predictions for other time points, such as 6 months or 2 years later, can be generated after collecting additional training data. (3) To reduce the computational burden, down-sampling was performed on the MRI scans, which may have caused the loss of some fine structural details. Future research could focus on generating high-resolution MRI scans. Although the generated images were compared with the ground truth images in terms of quality and quantity, further assessment from a clinical perspective is warranted—for example, predicting multi-term cognitive scores based on the generated images. (4) Experiments were performed only on the ADNI dataset. Expanding validation to other independent, multi-center cohorts would help verify the generalizability of the proposed model across diverse populations, imaging protocols, and clinical settings. Last but not least, brain morphology varies considerably between individuals. The progress map uses a linear deformation, which is insufficient to establish structural correspondence. Incorporating an elastic or diffeomorphic registration step or an additional deformation module could improve the precision of MRI change prediction in Alzheimer’s disease.

## Data Availability

The original contributions presented in the study are included in the article/supplementary material, further inquiries can be directed to the corresponding authors.

## Footnotes

1
http://dbm.neuro.uni-jena.de/vbm

2
https://fsl.fmrib.ox.ac.uk/fsl/fslwiki/FSL

3
https://www.tensorflow.org/

4
https://scikit-image.org/docs/stable/api/skimage.metrics.html
